# A simple approach to obtain hybrid Au-loaded polymeric nanoparticles with a tunable metal load†
†Electronic supplementary information (ESI) available. See DOI: 10.1039/c5nr06850a



**DOI:** 10.1039/c5nr06850a

**Published:** 2015-11-27

**Authors:** Edurne Luque-Michel, Ane Larrea, Celia Lahuerta, Víctor Sebastian, Edurne Imbuluzqueta, Manuel Arruebo, María J. Blanco-Prieto, Jesús Santamaría

**Affiliations:** a Department of Pharmacy and Pharmaceutical Technology , School of Pharmacy , University of Navarra , C/Irunlarrea 1 , E-31008 Pamplona , Spain . Email: mjblanco@unav.es ; Fax: +34 948 425 649 ; Tel: +34 948 425 600 ext. 6519; b IdiSNA , Fundación Instituto de Investigación Sanitaria de Navarra , Recinto del Complejo Hospitalario de Navarra. Calle Irunlarrea , 3. Pamplona 31008 , Spain; c Institute of Nanoscience of Aragon (INA) and Department of Chemical , Engineering and Environmental Technology , University of Zaragoza , C/Mariano Esquillor , s/n , I+D+i Building , 50018 , Zaragoza , Spain . Email: victorse@unizar.es ; Fax: +34 976 761879 ; Tel: +34 876555441; d Minimally Invasive Techniques Research Group (GITMI) , Universidad de Zaragoza , C\Miguel Servet , 177 , 50013 , Zaragoza , Spain; e CIBER de Bioingeniería , Biomateriales y Nanomedicina (CIBER-BBN) , Centro de Investigación Biomédica en Red , C/Monforte de Lemos 3-5 , Pabellón 11 , 28029 Madrid , Spain

## Abstract

A new strategy to nanoengineer multi-functional polymer–metal hybrid nanostructures is reported. By using this protocol the hurdles of most of the current developments concerning covalent and non-covalent attachment of polymers to preformed inorganic nanoparticles (NPs) are overcome. The strategy is based on the *in situ* reduction of metal precursors using the polymeric nanoparticle as a nanoreactor. Gold nanoparticles and poly(dl-lactic-*co*-glycolic acid), PLGA, are located in the core and shell, respectively. This novel technique enables the production of PLGA NPs smaller than 200 nm that bear either a single encapsulated Au NP or several smaller NPs with tunable sizes and a 100% loading efficiency. *In situ* reduction of Au ions inside the polymeric NPs was achieved on demand by using heat to activate the reductive effect of citrate ions. In addition, we show that the loading of the resulting Au NPs inside the PLGA NPs is highly dependent on the surfactant used. Electron microscopy, laser irradiation, UV-Vis and fluorescence spectroscopy characterization techniques confirm the location of Au nanoparticles. These promising results indicate that these hybrid nanomaterials could be used in theranostic applications or as contrast agents in dark-field imaging and computed tomography.

## Abstract 2

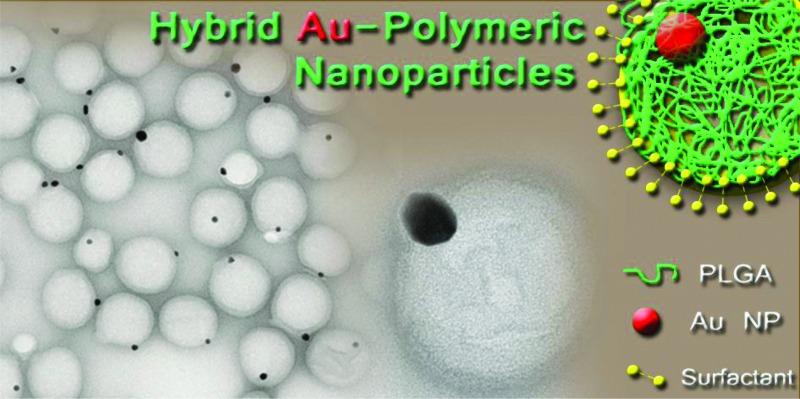
The production of Au–PLGA hybrid NPs with an exquisite control on the Au payload is described. The Au loading was achieved using the *in situ* reduction of gold precursors, obtaining a 100% encapsulation efficiency.

## Introduction

An important advance concerning the production of polymeric nanoparticles (NPs) is the ability to nanoengineer biocompatible polymer–metal hybrid nanostructures with remarkable optoelectronic and biomedical properties.^1^ Metal and metal oxide NPs, as well as quantum dots, can endow polymeric NPs with unique properties which make them potentially useful as Surface-Enhanced Raman Scattering (SERS) probes, contrast agents or advanced drug carriers.^2,3^ To date, most of the deve-lopments concerning polymer–hybrid nanomaterials that encapsulate metal NPs are based on covalent and non-covalent attachment^4^ of polymers to preformed inorganic NPs. Covalent attachment is achieved by the polymerization of monomers using mainly the “grafting-from”^5^ and the “*in situ* polymerization”^6^ techniques. In a typical “grafting-from” approach, polymer brushes are grown outwardly *in situ* from the functional surfaces of the preformed inorganic NPs by means of different surface-initiated polymerization techniques such as Atom Transfer Radical Polymerization (ATRP) and Reversible Addition–Fragmentation chain Transfer (RAFT). In the “*in situ* polymerization” approach, inorganic NPs are surface-functionalized and trapped in a “micelle reactor” containing the necessary chemicals such as the monomer and the catalyst. After initiating the polymerization, the inorganic NPs become trapped within the polymeric network. These strategies have proven to be effective in generating core/shell inorganic-polymer hybrids, but they typically require complex preparation procedures, including surface functionalization, a delicate control over the reaction conditions, and time-consuming purification processes. On the other hand, non-covalent immobilization requires the use of as-made polymers using the “layer by layer” (LBL),^7^ “direct encapsulation”^3,8–11^ and “template-assisted” approaches.^12–14^ The “layer by layer” procedure is based on the consecutive adsorption of alternating layers of positively and negatively charged polyelectrolytes on the inorganic NP surfaces, forming core/shell nanostructures. Although the main advantage of the LBL technique is its ability to precisely control the thickness of the polymer shell by controlling the number of polyelectrolyte layers, it is also a complex multi-step process and the shell often faces serious stability issues.^4^

The direct encapsulation approach consists of mixing preformed metal NPs separately prepared along with the oil or aqueous phase to produce an o/w or w/o/w emulsion, respectively. After synthesis, the organic phase is usually removed by solvent evaporation and then the polymeric NPs precipitate along with the encapsulated metal NPs. Although this one-pot procedure is easy to implement, the core encapsulation control is poor and its reproducibility limited, requiring a suitable core functionalization process if the core particles were dispersed in the organic phase.^15,16^ A new approach based on the direct encapsulation of gold (Au) NPs by the supercritical emulsion extraction (SEE) technique was successfully applied, obtaining sub-micron polylactic-Au particles (*ca.* 200 nm).^17^ However, although a good Au dispersion was achieved, the loading efficiency was limited (around 50%). Finally, the template-assisted approach is intrinsically a multi-step complex procedure, where preformed metal NPs are typically coated with a silica shell, which is in turn functionalized with certain groups that promote the growth of a polymeric layer. Afterwards, the removal of the sacrificial silica layer by an etching agent yields polymeric capsules that contain inner single NPs. Although the template-assisted approach is a promising way to create hybrid NPs with an excellent control on the loading of inorganic cores, it involves a rigorous regulation of the reaction conditions^18^ and a further template removal step that may introduce stability problems and/or induce chemical attachment on the NP cores.

Apart from the use of pre-formed NPs, a new strategy based on the *in situ* reduction of metal ions in a polymeric matrix circumvents some of the weaknesses of the previous techniques. The metallic ions are trapped in the polymeric particle and the reduction reaction is *in situ* activated by a reducing agent,^19^ UV-light^20^ or even ultrasounds.^21^ Although this is a more effective and lower-cost protocol than the previous one, the control on the loading and selective encapsulation of metal NPs must be still considerably improved.^10^ In addition, to the best of our knowledge, this technique has only been applied to the production of hybrid microparticles,^22,23^ but not to NPs. The latter require a higher degree of accuracy regarding encapsulation control especially whenever biomedical applications are considered, where the size of the carriers used in many applications is typically below 200 nm.^24,25^

These aforementioned procedures are not amenable to large scale production due to the lack of control during multi-step productions or due to the unavoidable formation of either polymer NPs without inorganic cores or inorganic NPs without a polymer coating in the one-pot approaches. It is widely recognized that controlling the size, morphology and payloads is one of the main barriers for the development of nanotechnology-based applications.^26^ Also, when a sufficiently large production per batch cannot be achieved, combining NPs derived from different batches may introduce unwanted variations in the quality and consequently affect the potential applications of the NPs.^27^ Therefore, it seems clear that developing a robust, scalable process to prepare polymeric NPs containing tunable inorganic NP payloads would be highly interesting. In particular, the preparation of monodisperse polymer NPs containing noble metal NPs represents a challenging objective with a multitude of potential applications.

Herein, we report a versatile one-pot protocol based on the *in situ* reduction to produce monodisperse poly(dl-lactic-*co*-glycolic acid), PLGA NPs smaller than 200 nm that bear either a single encapsulated Au NP or several smaller NPs with tunable sizes. Chloroauric acid and citrate ions were directly used as reagents and the PLGA polymer NPs as nanoreactors. The Au reagent dose within the polymer NPs, and consequently the Au NP size, was controlled by the formation of a double-emulsion with the polymer. *In situ* reduction of Au ions inside the polymeric NPs was achieved on demand by using heat to activate the reductive effect of citrate ions. In addition, we show that the loading of the resulting Au NPs inside the PLGA NPs is highly dependent on the surfactant used. To the best of our knowledge, this is the first case of a one-pot fabrication of highly monodisperse PLGA NPs with a tunable Au NP payload in their interior and an exquisite control over the Au NP loading. PLGA was chosen as the polymeric matrix because of its non-toxic character, biodegradability and high cellular uptake efficiency.^28^ PLGA undergoes hydrolysis in the presence of physiological water, releasing the original monomers which are easily metabolized in the body *via* the Krebs cycle without any systemic toxicity. On the other hand, Au NPs are widely used in a variety of biomedical applications, mainly on account of their optical properties.^29–31^ Here we use them as a practical example of the potential of the method developed to tailor the encapsulation of noble metal NPs in polymeric matrices for future use in theranostic applications or as contrast agents in dark-field imaging and computed tomography.

## Results and discussion

**Fig. 1:**
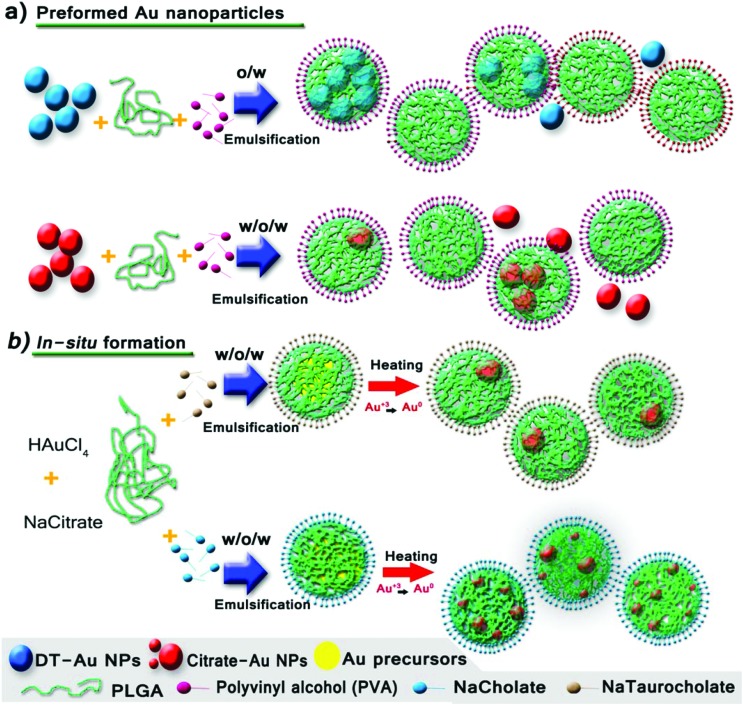
Schematic illustration of the synthesis of Au–PLGA hybrid NPs produced by: (a) direct encapsulation of the preformed hydrophilic (citrate) and hydrophobic (dodecanethiol – DT) Au NPs and (b) the in *situ* reduction method. The *in situ* reduction method was performed with different types of surfactants to tune the Au NP payload.

Two different alternatives to produce hybrid PLGA NPs were explored, involving respectively the encapsulation of preformed Au NPs (Fig. 1a) and the *in situ* formation of Au NPs inside the PLGA matrix from Au precursors (Fig. 1b).

### Encapsulation of preformed Au NPs – double w/o/w emulsion

**Fig. 2:**
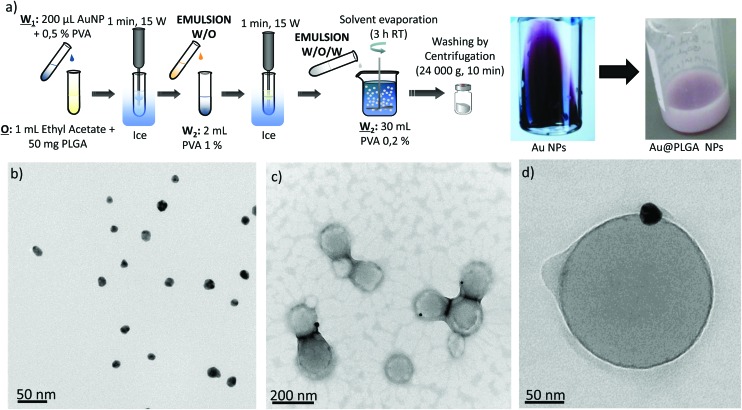
(a) Synthesis procedure of Au–PLGA hybrid NPs by direct encapsulation in w/o/w emulsion and visual appearance of the initial Au NPs and the Au–PLGA hybrids. TEM micrographs of: (b) Au-citrate NPs, (c) Au–PLGA hybrid NPs after solvent evaporation. (d) A detailed image of a Au–PLGA hybrid NP.

PLGA–Au NPs were first synthesized by an emulsion evaporation process following a direct encapsulation approach with preformed Au NPs (Fig. 1a and 2a). To this end, hydrophilic Au NPs were separately fabricated according to the well-known Turkevich method (Fig. 2b).^32^ Next, PLGA particles entrapping the previously synthesized Au NPs were prepared by a water in-oil-in-water (w/o/w) emulsion method followed by solvent evaporation of the volatile organic phase (ethyl acetate) at room temperature under stirring (Fig. 2a). Fig. 2d shows that for the PLGA NPs containing Au NPs, the Au position inside the PLGA matrix was near the surface. It was believed that this preferential location could be derived from the weak interaction of the hydrophilic Au particles with the organic phase in the w/o/w emulsion. It is likely that some citrate molecules located at the surface of Au NPs form hydrogen bonds between the hydroxyl groups of citrate and the surfactant used in the synthesis of PLGA NPs. On the other hand, the TEM characterization revealed a poor control of the Au NP loading, resulting in the presence of numerous PLGA NPs without a Au payload and Au NPs without a PLGA coating (Fig. 2c and S1a in the ESI†).

To improve the encapsulation of Au NPs, the concentration of Au NPs at the inner water phase utilized in the w/o/w emulsion was modified. As expected, the encapsulation efficiency increased as the concentration of Au NPs was increased, obtaining indifferently limited encapsulation efficiency (Fig. S1b in the ESI†). However, it was also observed that a highly concentrated Au colloid was not stable enough due to a concentration-polarization effect and the Au NPs tended to aggregate (Fig. S1b in the ESI†). This aggregation resulted in PLGA–Au NPs with a heterogeneous size distribution.

### Encapsulation of preformed Au NPs – single emulsion

**Fig. 3:**
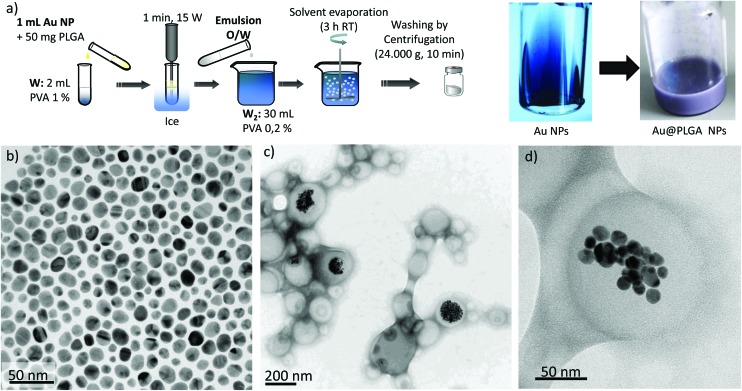
(a) Synthesis procedure of Au–PLGA hybrid NPs by direct encapsulation in o/w emulsion and visual appearance of the initial Au NPs and the Au–PLGA hybrids. TEM micrographs of: (b) DT-Au NPs, (c) Au–PLGA hybrid NPs after solvent evaporation. (d) A detailed image of a Au–PLGA hybrid structure with clustered Au NPs.

Since both the volume (200 μL) and the concentration of Au in the colloid used as an aqueous phase in the w/o/w emulsion had to be limited to get a stable emulsion, we also attempted the encapsulation of the Au NPs in a single o/w emulsion (Fig. 1a and 3a). To achieve this, the surface of the preformed Au NPs was functionalized with non-hydrophilic ligands using a previously described aqueous-to-organic phase transfer protocol (Fig. 3b).^33^ It is important to point out that the surface functionalization of Au NPs with dodecanethiol (DT) and their dispersion in ethyl acetate led to a dielectric change that resulted in the modification of the Surface Plasmon Resonance (SPR) peak and therefore in the optical properties of the NPs (Fig. S3a in the ESI†).^34,35^ Fig. 3a shows that, at the same concentration, the typical red-wine colour of the NPs produced by the Turkevich method in the water phase (see Fig. 2a) turns into a bluish colour after being surface-modified and dispersed in ethyl acetate. The DT-Au NPs were then encapsulated in PLGA by preparing an oil-in-water (o/w) emulsion followed by solvent evaporation of the volatile organic phase at room temperature under stirring (Fig. 3a). In this way, the DT-Au NPs were incorporated into the hydrophobic domain of PLGA molecules *via* hydrophobic interactions, and the PLGA NPs were then formed in the presence of polyvinyl alcohol (PVA) as emulsifier. The method produced hybrid structures with Au NPs located roughly in a central location inside the PLGA matrix (Fig. 3c and d). However, in spite of the fact that the single emulsification process facilitates the encapsulation of NPs due to the less restricted volume limitations between the aqueous/organic phases compared with the w/o/w method, the encapsulation yield achieved did not improve the results obtained by the double emulsion approach. In addition, Fig. 3c, d and S2† show that Au NPs tended to agglomerate and were unevenly distributed inside the PLGA NPs. The agglomerates encapsulated were constituted by a heterogeneous number of NPs, ranging from 10 to more than 100 units. This uncontrolled agglomeration inside the hybrid NPs prepared by the single emulsion method also gave rise to a non-homogeneous size distribution of Au PLGA NPs (207 ± 39 nm) and to the presence of empty PLGA NPs without metal NPs.

### *In situ* formation of Au NPs inside PLGA NPs

**Fig. 4:**
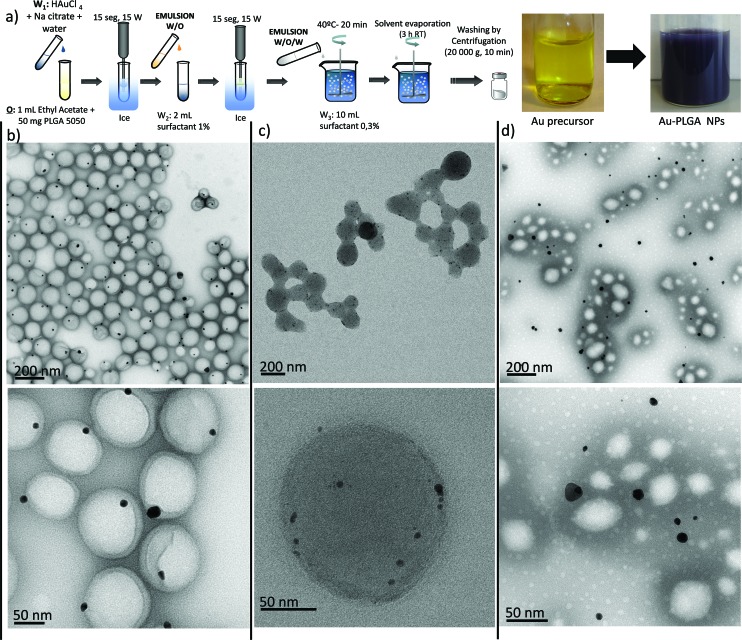
(a) Synthesis procedure of Au–PLGA hybrid NPs by the *in situ* reduction method in w/o/w emulsion and visual appearance of the initial Au NPs and the Au–PLGA hybrids. TEM micrographs of Au–PLGA NPs produced with different stabilizers: (b) taurocholate, (c) sodium cholate, (d) polysorbate 80.

Considering the problems already described (encapsulation efficiency, homogeneity of the NP load in the PLGA matrix) by the direct encapsulation approach, a new strategy was tested in which the nanoemulsification process was coupled to an *in situ* reduction method to generate Au NPs inside the PLGA NPs (Fig. 1b). In this case, PLGA NPs entrapping tetrachloroaurate and citrate ions were prepared by a double emulsion (w/o/w) method (Fig. 4a). This procedure was adopted because it could guarantee a similar load of Au^3+^ ions provided that the relative amounts of water and oil could be maintained in each PLGA NP. Similarly to a modified Turkevich procedure,^36^ the reduction of Au^3+^ ions to Au could be activated at the desired time by using a temperature increase, since at room temperature the Au^3+^ payload of each PLGA NP is stable enough to avoid reduction by citrate ions. Since PLGA 50 : 50 has a low vitreous transition temperature, 45–50 °C,^37^ 40 °C was selected as a suitable temperature to activate the redox reaction while keeping sufficient polymer rigidity to minimize the outward diffusion of the encapsulated chemicals. Then, the hybrid Au–PLGA NPs were formed by allowing evaporation of the organic phase at room temperature under stirring.

Three different types of surfactants, with different ionic natures, were selected to stabilize the double w/o/w emulsion: anionic sodium taurocholate (STC), anionic sodium cholate (SC), and non-ionic polysorbate 80. After 20 minutes of heating at 45 °C, the colour of the PLGA double emulsion prepared with STC as the surfactant gradually changed from pale yellow to dark purple. This colour change was a clear indication that the reduction of Au^3+^ ions was occurring. The UV-Vis spectra showed a noticeable peak associated with the surface plasmon resonance of Au NPs around 520 nm, supporting the Au NP growth (see Fig. S3b in the ESI†). Fig. 4b depicts the TEM micrograph of the Au–PLGA NPs formulation obtained with STC. It can be observed that every PLGA NP contains a single Au NP, as indicated by the dark sharp contrast associated with Au. A higher magnification is shown in Fig. 4b (below) where a single Au NP of 10 ± 2 nm in diameter was encapsulated in each PLGA NP, achieving a 100% selectivity in the encapsulation. This remarkable achievement confirms the effectiveness of the encapsulation of Au NP precursors. The containment of precursors was so efficient that Au reduction only occurred inside the PLGA NPs, using the PLGA micelle itself as a nanoreactor. On the other hand, the location of Au NPs was close to the surface of the PLGA NPs, as shown by the TEM images.

Finally, the loading of Au NPs by the procedure developed here was reproduced in 20 independent syntheses with nearly 100% reproducibility regarding the encapsulation of a single Au NP inside the PLGA NP (see Fig. S4 in the ESI†).

The type of surfactant applied in the formation of emulsions is a key variable for directing the production of PLGA NPs in a controlled manner.^38^ The changes in physico–chemical interactions between the hydrophobic and hydrophilic parts of the PLGA molecules produced by a change in the surfactant have an immediate effect on the final dimension of the resulting PLGA NPs and on the confinement of the Au precursor. Thus, when SC was used instead of STC during the double emulsion formation, the reduction of AuCl_4_^–^ by citrate ions gave rise to multiple encapsulated Au NPs with a size under 5 nm (Fig. 4c). The presence of SC directs the growth of Au^3+^ ions to tiny NPs randomly distributed in the PLGA matrix rather than producing a single, larger Au nanoparticle (Fig. 4).

The influence of the surfactant on the size of noble metal NPs has previously been described in the literature. It is well known that the stabilizers used in the reduction of Au^3+^ ions can control the size of the resulting Au NPs by lowering their high surface energy, leading to small Au NPs with strong stabilizers such as thiols and amines and to larger NPs with weaker stabilizers such as citrate ions.^35^ Although we do not have a direct measurement of the interaction, it can be speculated that the adsorption energy of SC is higher than the one of TSC. Again, the UV-Vis characterization showed a tiny SPR peak which is in full agreement with the Au NP size (see Fig. S3b in the ESI†), since when the size of Au NPs reaches dimensions below 5 nm their SPR band become very weak or even non-existent, because for these sizes the electron density in the conduction band becomes very small.^39^

**Fig. 5:**
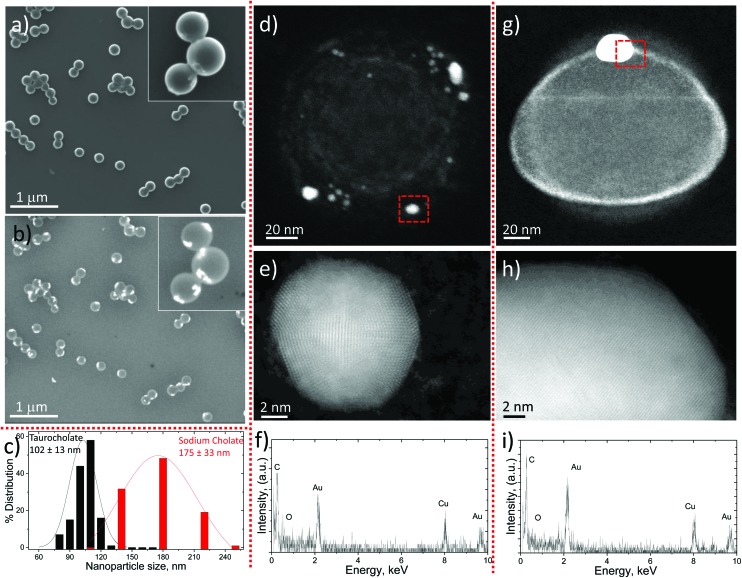
Au–PLGA hybrid NPs produced by the *in situ* reduction method in w/o/w emulsion with SC. (b) Back scattered electron micrograph to show the location of Au NPs in the same area as (a). (c) NP size distribution of Au–PLGA hybrid NPs produced with STC and SC as surfactants (data obtained from TEM analysis). (d) HAADF-STEM micrograph of representative Au–PLGA hybrid NP produced with SC to show by *Z*-contrast the location of Au NPs inside representative Au–PLGA NPs. (e) HAADF-HR STEM image to show the crystal fringes of an Au NP located inside a PLGA NP. (f) HAADF-EDS analysis of the area selected in (d) to confirm the presence of a Au NP. (g) HAADF-STEM micrograph of a representative Au–PLGA hybrid NP produced with STC to show by *Z*-contrast the location of the single Au NPs entrapped inside a PLGA NP. (h) HAADF-HR STEM image of the Au NP depicted in figure (g) to show the crystal fringes of the Au NPs located inside the PLGA NPs. (i) HAADF-EDS analysis of the area selected in (g) to confirm the presence of Au NPs.

SEM characterization using secondary and back-scattered electrons was performed in order to confirm the location of the Au NPs. Fig. 5a shows a SEM image obtained with secondary electrons where spherical PLGA NPs containing Au NPs are depicted. When the same PLGA NPs are analyzed through back-scattered electrons (Fig. 5b) the random presence of Au NPs, already observed by TEM, is clearly revealed. These pictures indicate that the location of Au NPs is close to the surface but they are still immersed in the PLGA matrix, in agreement with TEM results. Further evidence of the presence of the Au NPs inside the PLGA NPs will be provided by the experiments carried out with pyrene (see below).

Finally, polysorbate 80, a non-ionic surfactant, was also used to obtain Au–PLGA hybrid NPs. Compared to the anionic SC and STC surfactants, the use of polysorbate allows one to study the effect of surfactant polarity on the synthesis of hybrid Au–PLGA NPs by the *in situ* reduction method. Fig. 4d shows that polysorbate 80 neither stabilizes the PLGA molecules during the emulsification process nor the Au NPs during the *in situ* reduction process. It can be observed that PLGA NPs suffer from a lack of control of the shape and size after the solvent evaporation step and that a wide size distribution was obtained (Fig. 4d). On the other hand, most Au NPs were also located outside the PLGA NPs with an uncontrolled size distribution. These results imply that, in this case, the Au precursor and citrate ions were not properly encapsulated inside the double emulsion.

The above results support the double role played by the surfactant as a stabilizer of the polymer molecules during micelle formation and of the Au NPs during the *in situ* reduction process. In this study ionic surfactants (STC and SC) were preferred over nonionic ones (polysorbate 80) because the former could provide electrostatic stabilization to both polymeric and Au NPs. Also, the size of Au NPs loaded into PLGA NPs can be effectively tuned over 10 nm using STC or under 5 nm selecting SC. STC stabilizes the Au NPs inside the PLGA, likely due to the enhanced colloidal stabilization that the amino and the sulfur groups provide to the Au NPs. Electron-rich nitrogen donates electron density to the 5d and 6s orbitals of Au *via* its lone pairs. Au can also play the role of a proton acceptor and form nonconventional H-bonds with amine and hydroxyl groups,^40^ and strongly bonds to sulfur. It has been previously demonstrated that the S–Au bond is partially covalent (35%) and mostly electrostatic (65%).^41^ In addition, it is reported that both surfactants^42,43^ can provide electrons (reducing agent) to Au^3+^ ions through the hydroxyl groups and direct the formation of Au NPs. However, the reduction time required is higher than 4 days at room temperature. The higher electron-affinity between STC and Au NPs might be responsible for a successful controlled single Au NP encapsulation. The slow action of citrate and STC as weaker reducing agents also helps to avoid the formation of multiple single crystals. Thus, after the initial nucleation/reduction leading to the formation of crystal seeds, as Au^3+^ species are reduced, the concentration of reduced species does not reach the threshold required for a new nucleation, and instead they incorporate into growing crystals.

We found from a detailed analysis of the PLGA NPs’ size distribution that the PLGA NPs obtained with STC had the narrowest size distribution of the three tested after solvent evaporation, rendering a mean particle size with STC and SC of 102 ± 13 nm and 175 ± 33 nm, respectively (Fig. 5c). The corresponding mean NP sizes measured by Dynamic Light Scattering (DLS) in Au–PLGA hybrid NPs produced with STC and SC were 128 ± 6 nm (PDI 0.065 ± 0.006) and 197 ± 4 nm (PDI 0.101 ± 0.031), respectively. Notwithstanding some degree of agglomeration (slight in any case, as shown by the electron microscopy images). The observed differences in size between DLS and TEM may also arise from the drying process and the subsequent shrinkage that polymeric chains underwent during TEM sample preparation. It should be highlighted that according to previous reports, polymeric NPs with sizes less than 200 nm have shown great potential for both *in vitro* and *in vivo* applications.^24,25^ From the obtained results we can conclude that both SC and STC can stabilize Au NPs in the PLGA matrix to achieve 100% selectivity in the encapsulation by the double emulsion evaporation method with *in situ* reduction. Nevertheless, the different stabilization and reductibility during the nucleation-growth process of Au NPs enable the encapsulation of either big Au NPs (size > 10 nm) with STC or small Au NPs (size < 5 nm) with SC. On the other hand, PLGA NPs obtained with STC are more homogeneous in size and smaller than the ones produced with SC. Consequently, a balance between the stabilization of PLGA and Au atoms is required in the formation of Au–PLGA hybrid NPs.

A further microscopy analysis from a single Au–PLGA hybrid NP, using a STEM microscope with a HAADF detector sensitive to the atomic number, confirms the location of Au NPs close to the surface but embedded in the PLGA matrix (Fig. 5d and g). Although this observation was already inferred from the TEM images, in some cases there was some doubt as to whether the NPs were inside the PLGA particle or on its surface. It must be taken into account that the electron beam radiation that receives the PLGA NPs makes them shrink a few nanometers, and that may be enough to bring the Au NPs to the surface. This behavior can be observed in the halo which remains as shown in Fig. 5g after the shrinkage. Au NPs stabilized with SC are highly crystalline with the presence of twin defects as can be observed from the HR-STEM image in Fig. 5e. Similarly to Au NPs stabilized with SC, the use of STC gave rise to highly crystalline Au NPs (Fig. 5h). The energy-dispersive X-ray spectroscopy analysis (EDS) of the brightest NPs encapsulated in the hybrid Au–PLGA NPs produced with both surfactants confirms the presence of Au and not W crystals from the contrast agent, this could also be originated during the staining process and give a similar contrast (Fig. 5f and i).

**Fig. 6:**
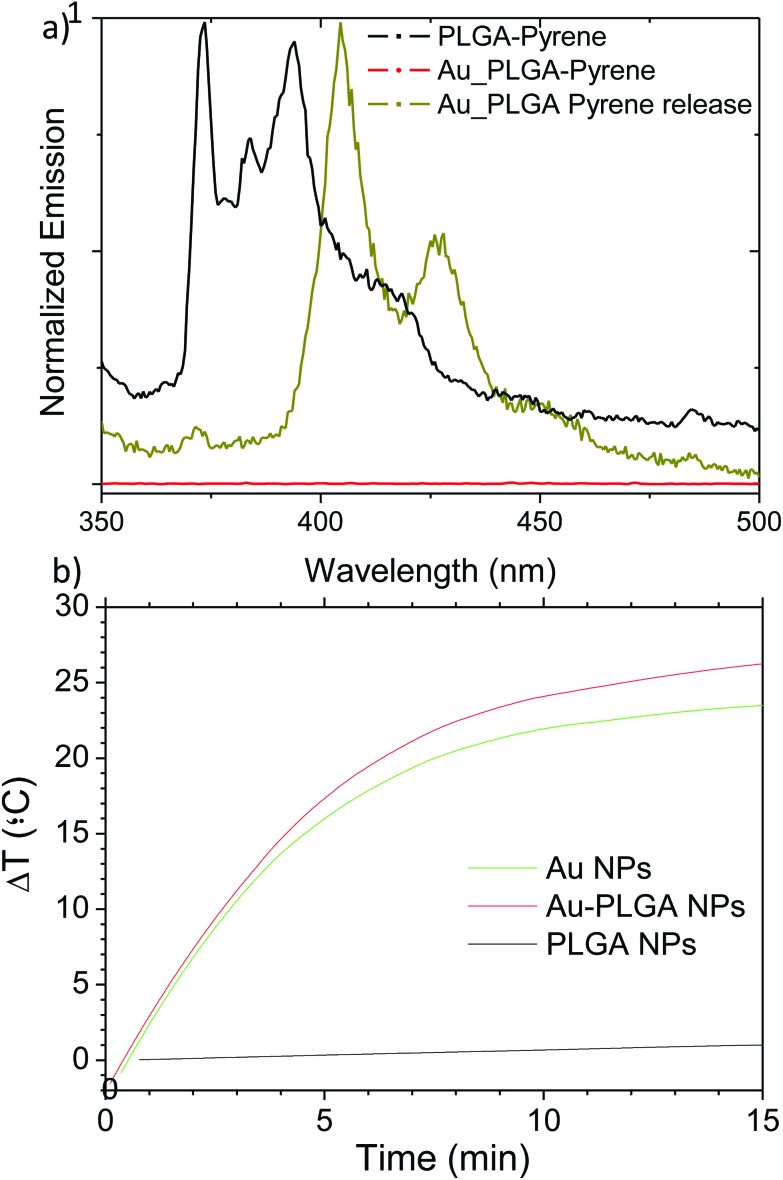
(a) Fluorescence spectra of pyrene: encapsulated in PLGA NPs, encapsulated in hybrid Au–PLGA NPs and dispersed in IPA after release from hybrid Au–PLGA NPs. [Au] 0.29 mg mL^–1^; [Pyrene] = 4.6 ppm. (b) Temperature increase as a function of time under laser irradiation (*λ* = 532 nm and power density of 4.2 W cm^–2^) of Au NPs, PLGA NPs and the hybrid Au–PLGA NPs. [Au] = 0.29 mg mL^–1^.

Pyrene is a photoactive fluorophore that can be considered as a good hydrophobic model molecule because of its low water solubility, well-defined fluorescence spectra, and a well-established method to statistically analyze its transfer rate from its carrier to the surrounding medium.^44^ Moreover, it is reported that the binding of pyrene to Au NPs renders organic–inorganic hybrid nanoassemblies suitable for light-harvesting and optoelectronic applications.^45,46^ We therefore used this system as a final direct proof of the presence of Au inside the PLGA matrix. Pyrene has different peak signals between 360 and 500 nm depending on solvent polarity. The intensity of the peaks in the absorption and emission spectra has often been used to sense the polarity of the microenvironment. Thus, the defined I/III emission intensity is usually related to the intensities of the peaks at 373 (*I*_1_) and 384 (*I*_3_) nm and it is used as a scale for solvent polarity.^47^ When pyrene is present in a polar solvent such as water, the I/III ratio equals 1.87; when pyrene is present in a non-polar solvent such as hexane, the I/III ratio equals 0.58.^47^ Fig. 6a shows the fluorescence spectrum of pyrene moieties encapsulated in PLGA NPs without Au. In this case the calculated I/III ratio equals 1.3, which corresponds to the ratio associated with ethyl acetate.^47^ Then, it can be inferred that pyrene moieties are located close to the PLGA polymer, where the ethyl acetate moieties, which still remain after the 3 h evaporation step, are adsorbed since the solubility of ethyl acetate in water is relatively low (8.3 g per 100 mL at 20 °C).

On the other hand, the fluorescence spectrum of the pyrene moieties encapsulated in the hybrid Au–PLGA NPs did not show the typical emission peaks of pyrene and no peaks were detected. This fact can be explained as a consequence of the quenching effect that Au NPs produce on pyrene due to the modification of electron and energy transfer processes, which deactivate the excited states of this fluorophore.^48^ Isopropyl alcohol (IPA) can be used to extract the pyrene from the pyrene-loaded PLGA NPs. We used this property to show that, in spite of the absence of the characteristic signals for the Au containing PLGA NPs (Fig. 6a), the hydrophobic interior of the PLGA NPs was loaded with pyrene. Indeed, after extraction with IPA the fluorescence signal of the solubilized pyrene was clearly shifted corroborating the presence of pyrene moieties associated with Au inside the PLGA NPs. This displacement could be an added advantage to the potential uses of the Au–PLGA hybrid NPs as biological tracers as well as in optoelectronic devices.

Since the UV-Vis characterization was able to show the interesting optoelectronic properties of the hybrid Au–PLGA nanoplatforms, they were exposed to continuous illumination to demonstrate that the Au NPs synthesized inside the PLGA matrix are crystalline enough to act as efficient light absorbers. The exposure of aqueous suspensions of Au–PLGA produced with SC to continuous illumination at a laser power of 4.2 W cm^–2^ (wavelength 532 nm) for 15 min resulted in an elevation of the dispersion mean temperature of *ca.* 26 °C (Fig. 6b), while a blank experiment with PLGA NPs without Au NPs inside showed temperature increases around 1 °C under the same conditions. On the other hand, a very similar heating (23 °C) was achieved when aqueous dispersions of Au NPs containing the same nominal Au loading (measured by ICP), 0.29 mg ml^–1^, were subjected to irradiation (Fig. 6b). These results clearly indicate that the Au encapsulated in the hybrid nanoplatforms are still efficient light absorbers. In addition, at this wavelength the attenuation by the PLGA shell was negligible and the hybrid Au–PLGA NPs achieved the same temperature elevation as a pure Au colloid. This may also be helped by the position of the Au NPs near the PLGA edge. The morphology of the hybrid Au–PLGA NPs after laser irradiation was conserved, as observed by TEM images (see Fig. S5 in the ESI†).

## Experimental

### Materials

The following chemicals were obtained from commercial suppliers and used as received: the polymer poly(d,l-lactic-*co*-glycolic acid) 50 : 50 (PLGA; *M*_W_ 38 000–54 000 Da, *T*_g_ 46–50 °C), under the commercial name of Resomer® RG 504 was purchased from Boehringer Ingelheim (Ingelheim, Germany) and Evonik Industries (Evonik Röhm GmbH, Germany). Sodium cholate, chloroauric acid (HAuCl_4_·3H_2_O), sodium citrate tribasic dehydrate ≥99.0% (Na_3_C_6_H_5_O_7_·2H_2_O), PVA (polyvinyl alcohol) *M*_W_ 85 000–124 000 Da, 97–99% hydrolyzed, taurocholic acid sodium salt hydrate ≥95% (TLC), ethyl acetate ACS reagent, dodecanethiol, phosphotungstic acid hydrate as a contrast agent for microscopy and fluorescent molecule pyrene ≥99.0% were purchased from Sigma Aldrich (St Louis, MO, USA).

### Synthesis of Au NPs

Citrate-capped Au NPs were synthesized according to the Turkevich method but by decreasing the synthesis temperature,^32^ using HAuCl_4_ as a Au precursor and sodium citrate as both reducing agent and stabilizer. Briefly, 100 ml of a HAuCl_4_ solution containing 2.9 mg of Au was added to 50 ml of distilled water and kept at 70 °C. Afterwards, 5 mL of 1% sodium citrate solution was added while stirring vigorously. After continuous stirring for 30 minutes, the dispersion was allowed to cool down.

The dodecanethiol (DT)-capped Au NPs were synthesized using the previously prepared citrate capped Au NPs and using a phase transfer ligand exchange method.^33^ Citrate-Au NPs at high concentrations (2 × 10^–8^–5 × 10^–8^ M) in water were put into contact with DT. After addition of acetone, NPs were extracted into DT by swirling the solution for a few seconds, upon which the aqueous phase became clear, indicating that no Au NPs remained. Excess DT was removed by diluting the DT coated Au NPs in ethyl acetate and performing several centrifugation cycles.

### Synthesis of hybrid Au–PLGA NPs by direct encapsulation using a double-emulsion w/o/w

Au NPs were encapsulated into PLGA NPs by the water-in-oil-in-water emulsion solvent evaporation method. Briefly, an aqueous phase composed of 200 μL Au colloid NPs (4 mM–50 mM) and PVA (0.5% w/v) was mixed by ultrasonication (15 W, 1 min, Branson Sonifier 450, Branson Ultrasonics Corp., Danbury, USA) with the organic phase containing 50 mg of PLGA dissolved in 1 mL of ethyl acetate. This w/o emulsion was emulsified again by ultrasonication (15 W, 1 min) with 2 mL of a 1% (w/v) PVA aqueous solution to form a w/o/w emulsion. This final emulsion was then poured into a 30 ml solution of 0.2% (w/v) PVA and continuously stirred for at least 3 h at room temperature to allow solvent evaporation and NP formation. Particles were collected by centrifugation (24 000*g*, 15 min) (Sigma Laboratory Centrifuges, 3K30, Rotor no. 12150-H, Osterode am Harz, Germany) and washed three times with ultrapure water.

### Synthesis of hybrid Au–PLGA NPs by direct encapsulation using a single-emulsion o/w

DT functionalized Au NPs were encapsulated into PLGA NPs by the oil-in-water emulsion solvent evaporation method. Briefly, 50 mg of PLGA was dissolved in 1 mL of DT-Au NPs (4 mM–50 mM) dispersed in ethyl acetate. This organic phase was emulsified with 2 ml of a 1% (w/v) PVA aqueous solution by ultrasonication at 15 W for 1 min in an ice bath. The formed o/w emulsion was then poured into a 30 mL solution of 0.2% (w/v) PVA and continuously stirred for at least 3 h at room temperature to allow solvent evaporation and NP formation. Particles were collected by centrifugation (24 000*g*, 15 min) and washed three times with ultrapure water.

### Synthesis of hybrid Au–PLGA NPs by *in situ* reduction using a double-emulsion w/o/w

Au NP precursors were encapsulated into PLGA NPs by the double emulsion solvent evaporation method. Once the emulsion was formed, the temperature was increased to activate the reduction of the Au precursor by sodium citrate. Briefly, 5 mg of Au(iii) chloride hydrate and 25 mg of sodium citrate tribasic dihydrate were dissolved in 50 μL of MilliQ water. This aqueous phase was emulsified with 1 mL of ethyl acetate containing 50 mg of PLGA by ultrasonication (Branson Sonifier 450, Branson Ultrasonics Corp., Danbury, CT, USA) at 30% amplitude for 15 seconds in an ice bath. The formed w/o emulsion was emulsified likewise with 2 ml of a 1% (w/v) surfactant aqueous solution (sodium cholate, taurocholate or polysorbate 80) to obtain a w/o/w emulsion that was added into a 10 ml of 0.3% (w/v) surfactant solution. Then to promote the reduction of Au^3+^ ions to Au^0^ and consequently the formation of Au NPs inside the PLGA NPs, the temperature was increased to 45 °C for 20 minutes in a closed vessel to avoid solvent evaporation. Finally the vessel was opened and the formulation was stirred for at least 3 h at room temperature to allow solvent evaporation. Particles were collected by centrifugation (20 000*g*, 10 min) and washed three times with ultrapure water.

### Characterization

The UV-Vis spectra of Au NPs and Au–PLGA hybrid NPs were measured using a Jasco V-670 spectrophotometer. The fluorescence spectra of the hybrid NPs containing pyrene were measured using a fluorimeter (Perkin-Elmer LS55). Average particle size and size distributions were determined by DLS with a particle size analyzer (Zeta Plus, Brookhaven Instruments Corporation, NY) at a fixed angle of 90° at room temperature. The surface morphology of the nanocomposites was investigated by using a FE-SEM (Inspect F-50, FEI, Eindhoven) at an accelerating voltage of 10–15 kV. The NPs were immobilized on a silicon chip, stained with phosphotungstic acid hydrate, dried and coated with a platinum layer. Preliminary transmission electron microscopy observations were carried out at the LMA-INA-Universidad Zaragoza facilities using a T20-FEI microscope with a LaB6 electron source fitted with a “SuperTwin®” objective lens allowing a point-to-point resolution of 2.4 Å. Aberration corrected scanning transmission electron microscopy (Cs-corrected STEM) images were acquired using a high angle annular dark field detector in a FEI XFEG Titan electron microscope operating at 300 kV and equipped with a CETCOR Cs-probe corrector from the CEOS Company allowing the formation of an electron probe of 0.08 nm. Elemental analysis was carried out with an EDS (EDAX) detector which allows performing EDS experiments in the scanning mode. A 2.5 μL suspension of stained hybrid Au–PLGA NPs was pipetted onto a TEM copper grid with a holey carbon film. Samples were allowed to evaporate completely and then analyzed.

Irradiation experiments were performed with a collimated VIS light at 532 nm and a power density of 4.2 W cm^–2^. Measurements were carried out in 24-cell culture insert plates made of polycarbonate and with a fixed volume of 1 ml of dispersion. The distance between the laser head and the sample was approximately 1 cm. In all the assays the sample concentration used was 0.29 mg ml^–1^ of Au and 5 mg ml^–1^ of PLGA.

## Conclusions

Novel hybrid Au–PLGA NPs under 180 nm in size have been synthesized by coupling the nanoemulsion and the *in situ* reduction techniques in a one-pot procedure. This protocol enables the complete encapsulation of Au NPs inside PLGA NPs, which is much more efficient that the direct encapsulation method that has usually been employed so far. In addition, the Au load distribution (as a single NP or multiple smaller NPs) can be tailored by the appropriate choice of a surfactant. The electrostatic nature of the surfactants used and the presence of electron-rich nitrogen and sulfur in the STC structure are deemed to be responsible for the successful stabilization and consequent encapsulation. The presence of Au NPs close to the surface but still inside the PLGA matrix was confirmed by electron microscopy observations (SEM, TEM, STEM), laser irradiation and pyrene fluorescence tracking. Also this protocol can potentially be extrapolated to other reduction reactions used for the synthesis of metal NPs at low temperatures. Interestingly, the developed NPs could be used in theranostic applications or as contrast agents in dark-field imaging and computed tomography.
