# Supplementary material for: A simple approach to obtain hybrid Au-loaded polymeric nanoparticles with a tunable metal load
Source: Nanoscale. 2015 Nov 27;8(12):6495–506. doi: 10.1039/c5nr06850a (PMC4819683; doi:10.1039/c5nr06850a)
Supplement: Supplementary file 1 [file NR-008-C5NR06850A-s001.pdf]

Electronic Supplementary Material (ESI) for Nanoscale.
This journal is © The Royal Society of Chemistry 2015

A simple approach to obtain hybrid Au-loaded polymeric nanoparticles with tunable metal load.

Edurne Luque-Michel, Ane Larrea, Celia Lahuerta, Víctor Sebastian , Edurne Imbuluzqueta, Manuel Arruebo , María J. Blanco-Prieto and Jesús Santamaría .

Supporting Information

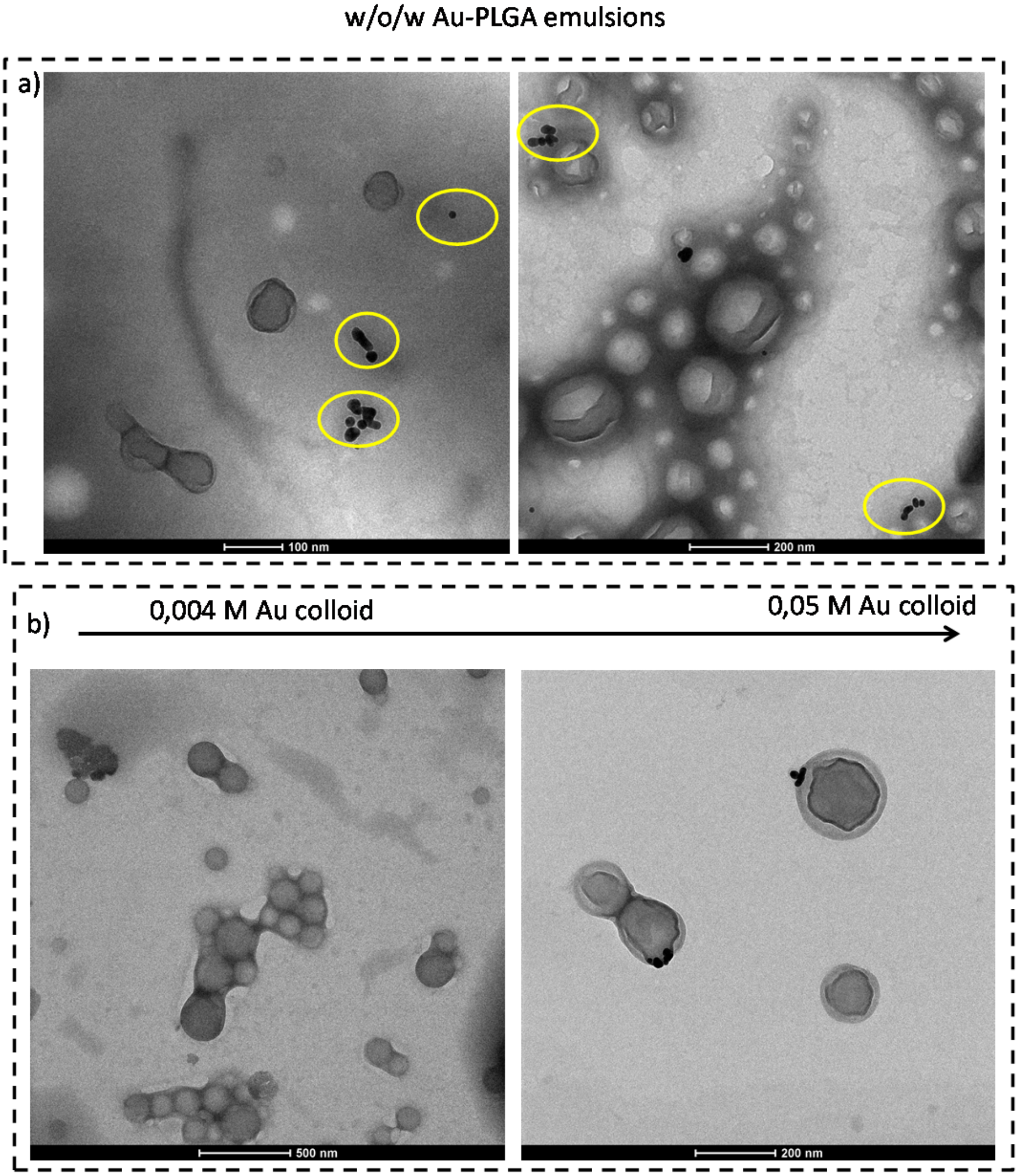

Figure S1.- TEM micrographs of Au-PLGA hybrid nanoparticles produced by the direct encapsulation method in w/o/w emulsion. a) Marked areas show the location of Au-NPs outward from the PLGA-NPs; b) Au aggregation as the concentration of Au colloid is increased.

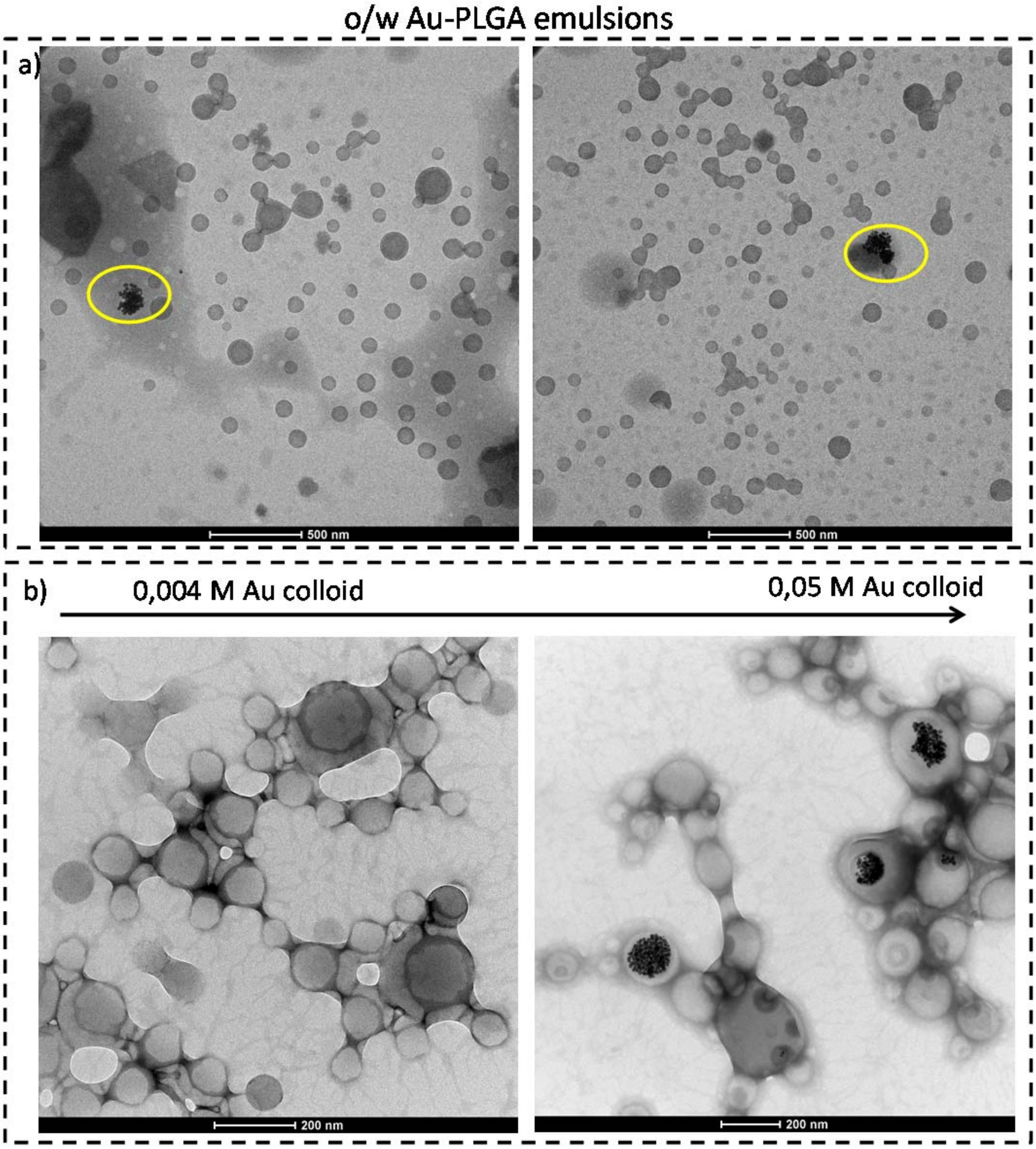

Figure S2.- TEM micrographs of Au-PLGA hybrid nanoparticles produced by the direct encapsulation method in o/w emulsion. a) Marked areas show the location of Au-NPs outward from the PLGA-NPs; b) Au aggregation as the concentration of Au colloid is increased.

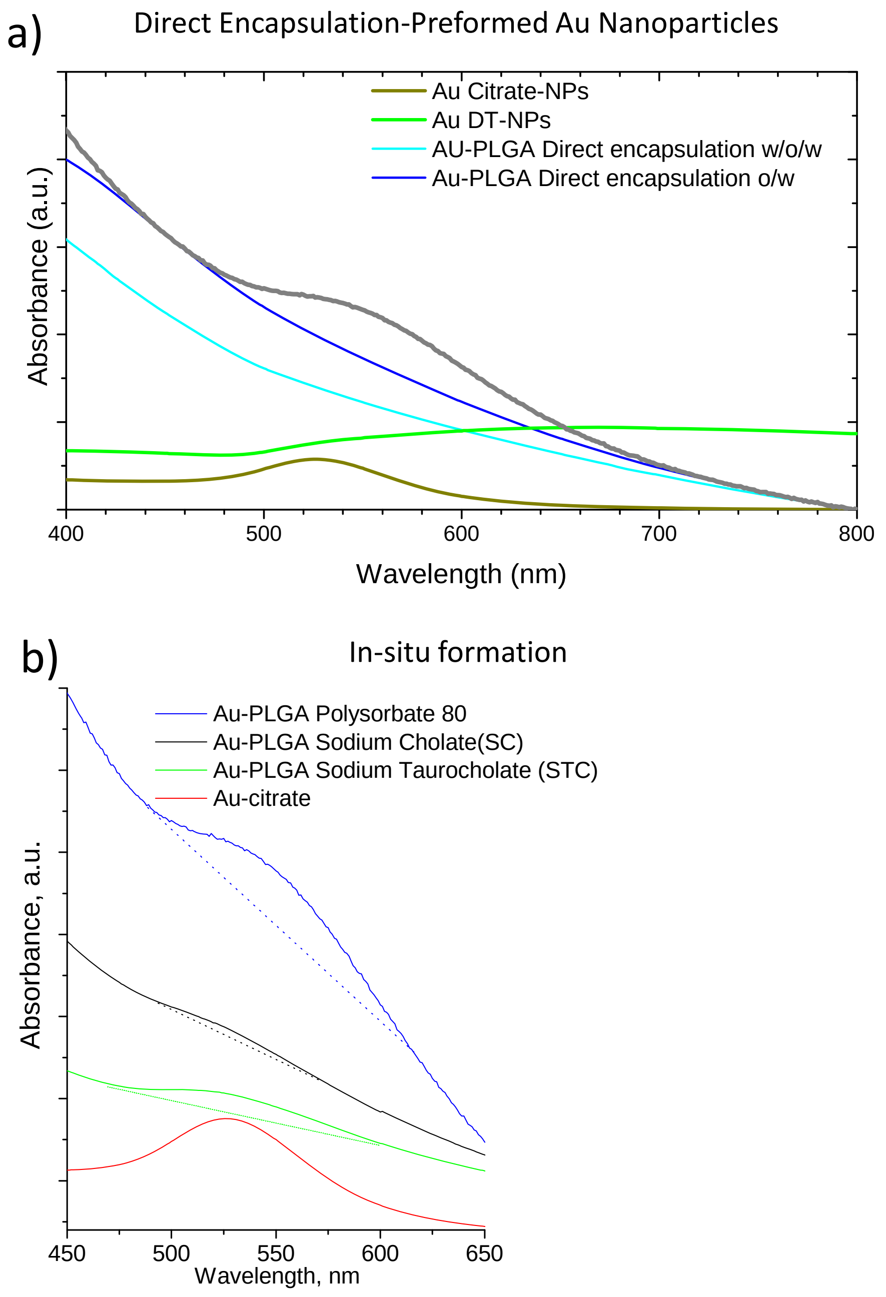

Figure S3.- UV-VIS spectra of the Au-NPs and Au-PLGA NPs produced in this work: a) Direct encapsulation approach, b) *In-situ* reduction.

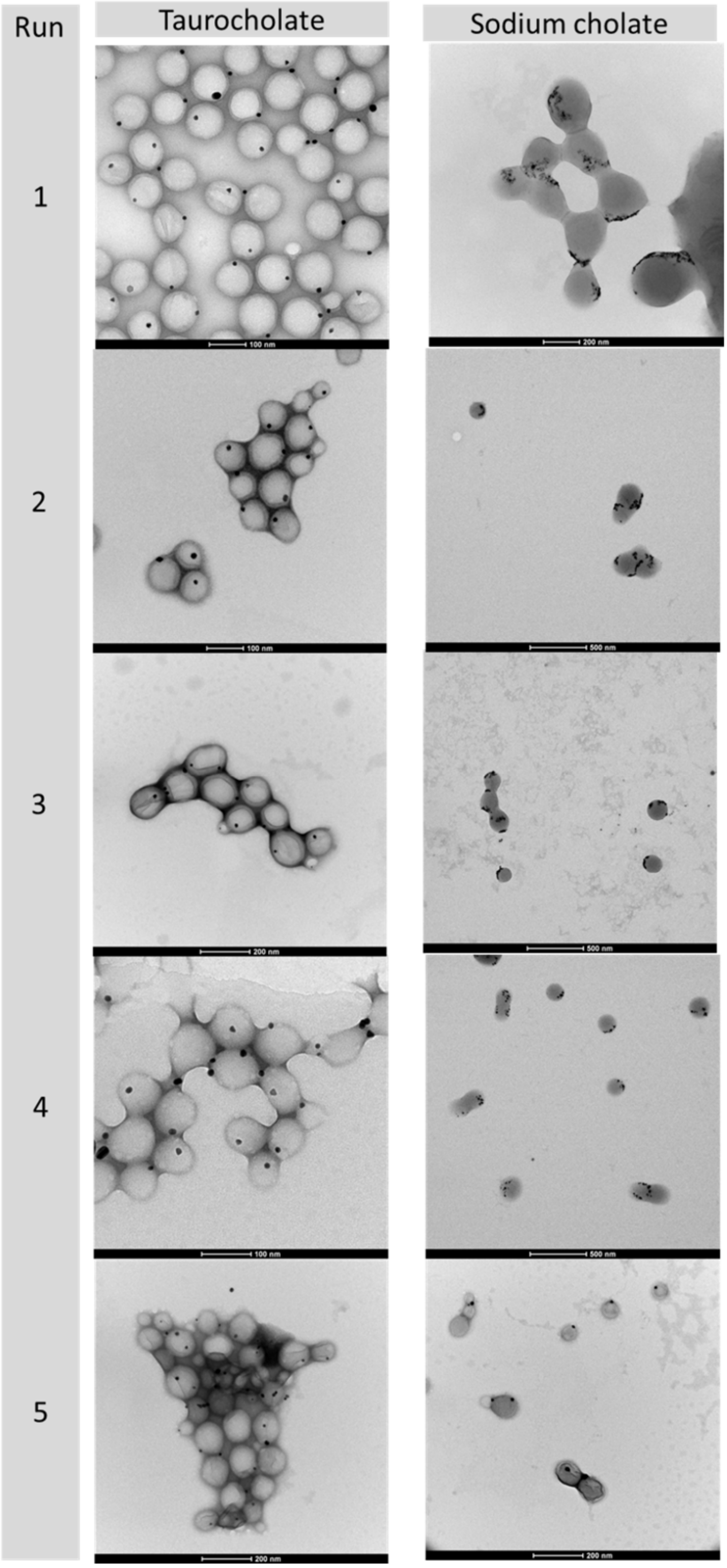

Figure S4.- TEM micrographs of Au-PLGA hybrid nanoparticles produced by the *in-situ* reduction method in w/o/w emulsion with STC and SC in 5 different batches to show the synthesis procedure reproducibility.

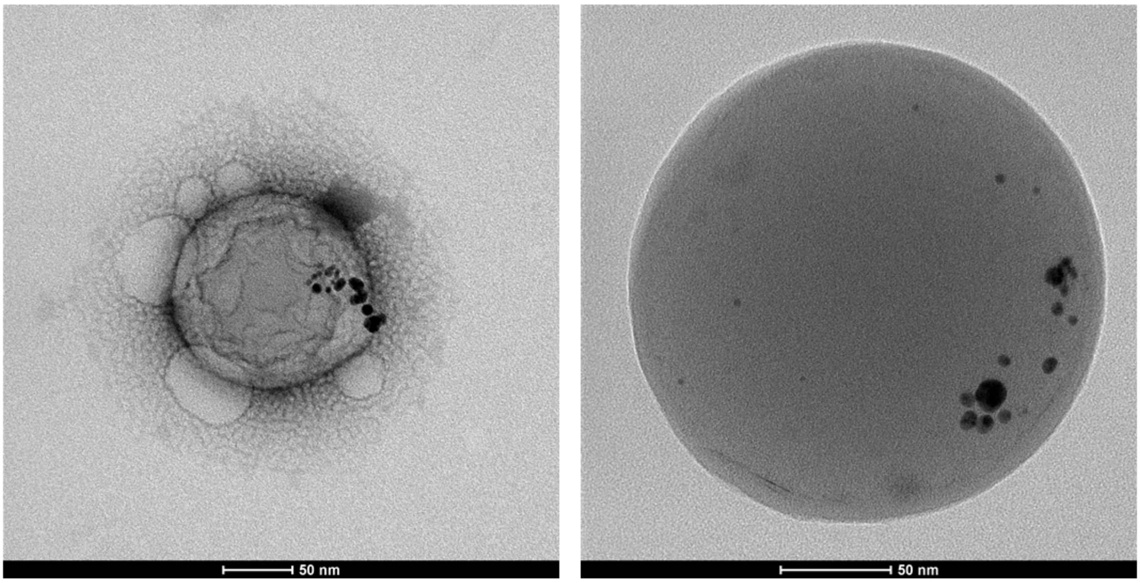

Figure S5.- TEM micrographs of Pyrene- Au-PLGA hybrid nanoparticles produced by the "in-situ reduction method" in w/o/w emulsion with SC after laser radiation for 15 min.
